# Diagnostic effect of shear wave elastography imaging for differentiation of malignant liver lesions: a meta-analysis

**DOI:** 10.1186/s12876-019-0976-2

**Published:** 2019-04-25

**Authors:** Xing Hu, Xiaojie Huang, Hui Chen, Tong Zhang, Jianhua Hou, Aixin Song, Lei Ding, Weiyuan Liu, Hao Wu, Fankun Meng

**Affiliations:** 1 Ultrasound and Functional Diagnosis Center, Beijing Youan Hospital, Capital Medical University, No 8, Xitoutiao, Youanmenwai, Fengtai District, Beijing, 100069 China; 2 Center for Infectious Disease, Beijing Youan Hospital, Capital Medical University, No 8, Xitoutiao, Youanmenwai, Fengtai District, Beijing, 100069 China; 3 School of Biomedical Engineering, Capital Medical University, Beijing, China

**Keywords:** Liver neoplasms, Ultrasonography, Elasticity imaging techniques, Diagnosis, Meta-analysis

## Abstract

**Background:**

Shear wave elastography (SWE) imaging have been proposed for characterization of focal liver lesions. We conducted a meta-analysis to evaluate the accuracy and clinical utility of SWE imaging for differentiation of malignant and benign hepatic lesions.

**Methods:**

PubMed, Embase, Web of Science, and the Cochrane Library were systematically reviewed to search for studies published between January 1, 1990, and November 30, 2018. The studies published in English relating to the evaluation the diagnostic accuracy of SWE imaging for distinguishing malignant and benign liver lesions were retrieved and examined for pooled sensitivity, specificity, likelihood ratios, and diagnostic odds ratios, using bivariate random-effects models. The hierarchical summary receiver operating characteristic (HSROC) curve was estimated to assess the SWE imaging accuracy. The clinical utility of SWE imaging for differentiation of malignant liver lesions was evaluated by Fagan plot.

**Results:**

A total of 15 studies, involving 1894 liver lesions in 1728 patients, were eligible for the meta-analysis. The pooled sensitivity and specificity for identification of malignant liver lesions were 0.82 (95% CI: 0.77–0.86) and 0.82 (95% CI: 0.76–0.87), respectively. The AUC was 0.89 (95% CI: 0.86–0.91). When the pre-test probability was 50%, after SWE imaging measurement over the cut-off value (positive result), the corresponding post-test probability for the presence of malignant liver lesions was 82%; the post-test probability was 18% after negative measurement.

**Conclusions:**

SWE imaging showed high sensitivity and specificity in differentiating malignant and benign liver lesions and may be promising for noninvasive evaluation of liver lesions.

**Trial registration:**

The review was registered in the International Prospective Register of Systematic Reviews (PROSPERO): CRD42018104510.

**Electronic supplementary material:**

The online version of this article (10.1186/s12876-019-0976-2) contains supplementary material, which is available to authorized users.

## Background

The development and extensive application of imaging technology has resulted in increased detection of focal liver lesions (FLLs) [1]. Liver cancer is the second most common cause of death from cancer worldwide [2]. Therefore, it is crucial to differentiate malignant from benign liver lesions, despite how extremely challenging that might be amid the wide variation of FLLs.

Ultrasonography is commonly used as the first imaging technique for detecting and distinguishing focal liver lesions because of its availability, low cost, and safety. Contrast-enhanced ultrasound (CEUS) has been shown to be a practicable and accurate method, because it can increase the sensitivity and specificity of detection of focal liver lesions detected by ultrasound to above 90 and 80%, respectively [3, 4]. However, some nodules are still difficult to distinguish, especially in the context of liver cirrhosis; moreover, the adverse effects of contrast agents limits the use of this technology to some extent. Liver biopsy has always been regarded as the gold standard for differentiating malignant and benign lesions. Despite its strengths, liver biopsy is an invasive procedure and could give rise to several complications such as pain, bleeding, and risks of mortality [5, 6].

Ultrasound elastography (USE) is a noninvasive method for the determination of tissue stiffness and the measurement value is usually altered by specific pathological or physiological processes of soft tissues (such as malignancy, inflammation, et al) [7]. Quantitative ultrasound elastography methods currently include acoustic radiation force impulse (ARFI) and transient elastography (TE) techniques [8]. The term “shear wave elastography” (SWE) refers to the technique of detecting shear-wave velocity (SWV) excited by acoustic radiation forces [9]. Both point shear-wave elastography (pSWE) and two-dimensional shear-wave elastography (2D-SWE) rely on the ARFI technique, which uses focused, short-duration acoustic pulses to deform localized tissue and generate shear waves [10]. Although both pSWE and 2D-SWE use ARFI to generate shear waves, pSWE is often referred to as ARFI elastography in some literature and 2D-SWE is referred to as real-time two-dimensional SWE (RT-2D-SWE).

A series of studies evaluate the performance of USE in quantifying tumor stiffness to characterize focal liver lesions [11–13]. In this study, we performed a systematic review and meta-analysis to assess the diagnostic accuracy and clinical utility of SWE imaging in differentiating malignant and benign FLLs.

## Methods

The review was registered in the International Prospective Register of Systematic Reviews (PROSPERO, http://www.crd.york.ac.uk/PROSPERO): CRD42018104510. We reported this study in accordance with the Preferred Reporting Items for Systematic Reviews and Meta-analyses (PRISMA) of Diagnostic Test Accuracy Studies [14].

### Literature search

We searched PubMed, Embase, Web of Science, and the Cochrane Library for studies published between January 1, 1990, and November 30, 2018, to identify articles evaluating SWE for distinguishing malignant and benign liver lesions. The following search strategy including Medical Subject Heading (MeSH) terms and a series of relevant keywords was used: ((liver lesion) OR (liver neoplasm) OR (liver cancer) OR (hepatic lesion) OR (hepatic tumor)) AND ((shear wave elastography) OR (SWE) OR (acoustic radiation force impulse) OR (ARFI) OR (virtual touch tissues quantification) OR (VTQ) OR (ultrasound elastography)) AND ((diagnosis) OR (differentiation) OR (evaluation) OR (distinguishing) OR (discriminate)). We also retrieved the reference lists of related studies manually and searched for other studies that might be omitted in electronic retrieval. The search was limited to journal articles written in English.

### Selection criteria

The included studies were required to fulfill the following inclusion criteria: (1) evaluated the performance of SWE imaging for differentiation of malignant and benign liver lesions; (2) used an appropriate reference standard for the diagnosis, such as cytology/histology acquired by biopsy or surgical specimens, or clinical imaging findings (CEUS or computed tomography/magnetic resonance imaging [CT/MRI]); (3) reported data sufficient to calculate the diagnostic accuracy results of SWE imaging (true positive, false positive, false negative, and true negative) for distinguishing liver lesions. The appropriate author was contacted by e-mail if such data were unavailable, and the study would be excluded if no author’s reply. (4) The study included at least 30 patients for the purpose of attaining good reliability. Studies were excluded if they were in a language other than English or were animal experiments. Only the most recent study could be included if the publications used an overlapping cohort of patients.

### Data extraction and quality assessment

The studies were retrieved and assessed independently by two reviewers; conflicts were resolved by consulting with a third investigator. The data were extracted independently by two investigators according to the predefined protocol. The following data were extracted from included studies: author, year of publication, country, study design, elastography modality, ultrasonic instrument, number of patients, number of liver lesions, number of the malignant liver lesions, invalid measures, lesion types, nodule size, reference standard for the diagnosis, proportion of cirrhosis, proportion of chronic liver disease, and the cut-off values. In addition, true positive (TP), false positive (FP), false negative (FN), and true negative (TN) could be extracted directly or calculated indirectly. Two investigators independently assessed the quality of the included studies by the Quality Assessment of Diagnostic Accuracy Studies 2 (QUADAS-2) [15], with divergences resolved by consensus.

### Statistical analysis

Summary measures of the SWE imaging accuracy (sensitivity, specificity, likelihood ratios, and diagnostic odds ratios) were calculated using a bivariate random-effects model. A hierarchical summary receiver operating characteristic (HSROC) curve was also plotted, and the area under the ROC curve (AUC) was calculated using bivariate model. The inconsistency index (I^2^) and Cochrane Q statistic were used to estimate the heterogeneity across studies. I^2^ value greater than 50% or a *P* value less than 0.10 suggested substantial heterogeneity. Sensitivity analysis was performed by removing studies in which the cut-off values of shear wave velocity (SWV) were less than 2.0 m/s to estimate whether undue influence of a single study was possible.

In addition, univariate meta-regression analyses were performed to explore the sources of potential heterogeneity among studies. The covariates included the following: elastography modality (pSWE vs. 2D-SWE), study location (Asian vs. European or North American), gold standard (histopathology only vs. histopathology and/or others), number of liver lesions (≥100 vs.<100), prevalence of malignant liver lesions (≥50% vs. < 50%), blinded from the results of the reference standard before interpretation of SWE (blinded vs. not blinded), and attrition rate (≥10% vs.<10%). Furthermore, groups were divided into subgroups based on the heterogeneity between studies. We performed a separate analysis of the studies that reported the cut-off value of the SWV/elasticity in FLL, as well as the cut-off value of the SWV/elasticity ratio (FLL to surrounding liver parenchyma).

We performed the Fagan plot to assess the clinical utility of the SWE imaging [16]. We calculated pre-test probabilities of 25, 50, and 75% versus corresponding post-test probabilities, following a positive or negative measurement of SWE. The potential publication bias was inspected by examining a Deeks funnel plot asymmetry test, with *P* < 0.1 for the slope coefficient indicating significant asymmetry [17].

All statistical analyses were conducted using Stata version 13.0 (StataCorp, College Station, TX) with midas and metandi modules.

## Results

### Search results and study characteristics

**Fig. 1:**
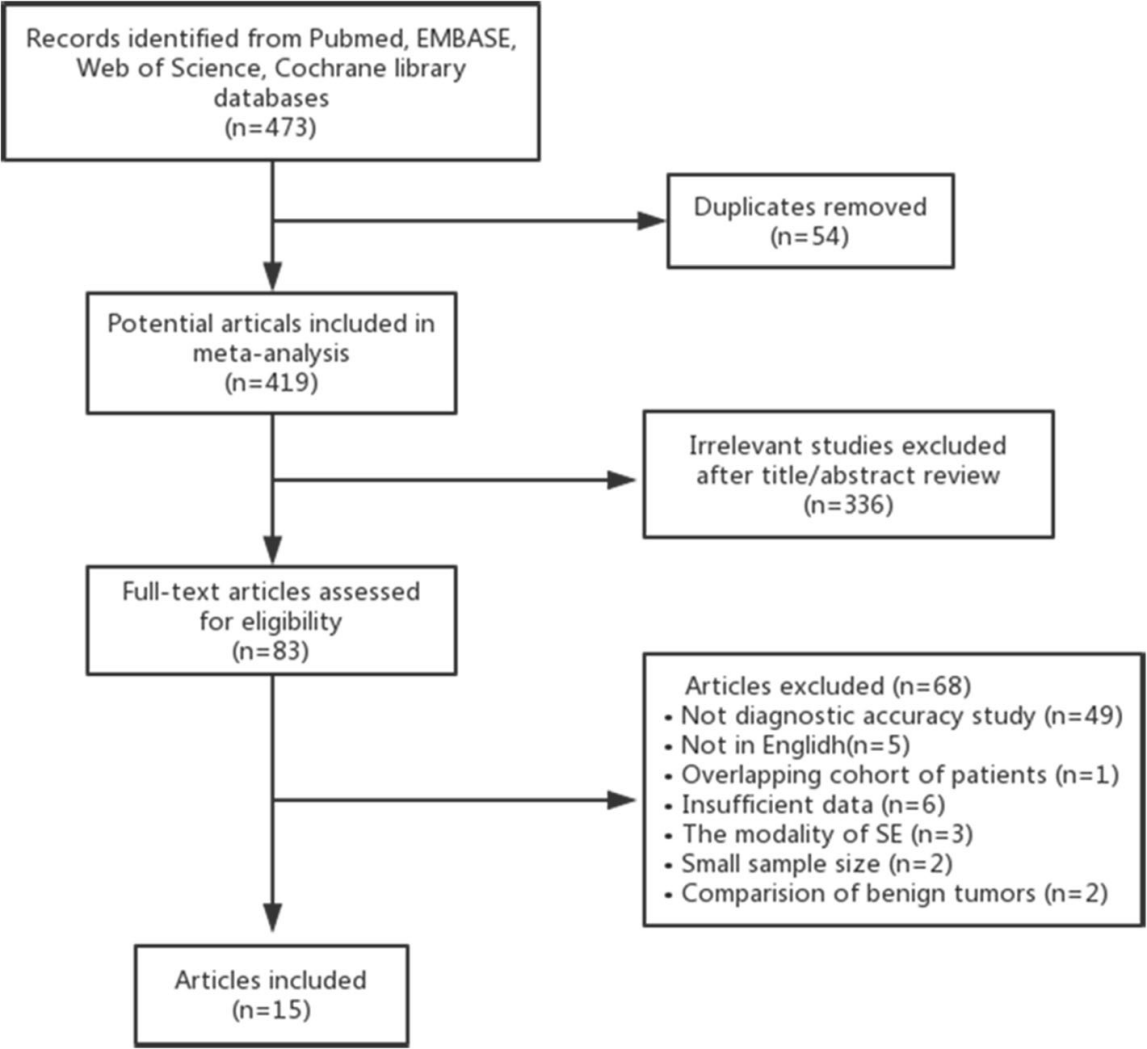
Flow chart of study selection process

Based on the predefined search strategies, a total of 473 studies were retrieved initially, with 54 duplicates. After eliminating 336 irrelevant studies on review of the titles and abstracts, 83 potentially relevant studies underwent full-text review to determine their eligibility for inclusion. Sixty-eignt articles were excluded for the following reasons: not diagnostic accuracy study (*n* = 49), not in English (*n* = 5), overlapping cohort of patients (*n* = 1), insufficient data (*n* = 6), the modality of strain elastography (*n* = 3), small sample size (*n* = 2), and focus on the comparison between benign tumors (n = 2) [13, 18]. Finally, 15 articles that fulfilled the inclusion criteria were included in this meta-analysis. The flow chart of the study selection appears in Fig. 1.

The characteristics of the 15 included studies are summarized in Table 1 and Additional file 1: Table S1. A total of 1894 liver lesions (673 benign, 1221 malignant) in 1728 patients were investigated. The cut-off value of shear wave speed in pSWE ranged from 1.82 m/s to 2.5 m/s, the cut-off value of Young modulus in 2D-SWE ranged from 20.7 Kpa to 24.43 Kpa, and the cut-off value of SWV/elasticity ration (FLL to surrounding liver parenchyma) ranged from 1.3 to 1.67. All of the 15 included studies were prospective in design. Twelve studies [19–30] used pSWE as the diagnostic imaging modality; the other 3 studies [31–33] performed 2D-SWE imaging modality. Ten studies [19, 20, 22, 23, 26, 28, 29, 31–33] exclusively reported the cut-off value of the SWV/elasticity in FLL, whereas 4 studies [21, 24, 25, 30] reported the cut-off value of the SWV/elasticity and the cut-off value of the SWV ratio. In addition, 1 study [27] reported the sum of the SWV (FLL and surrounding liver parenchyma). There were respectively four articles [22, 23, 30, 33] and six articles [19, 20, 23, 26, 30, 31] reporting the proportion of patients with cirrhosis and the proportion of chronic liver disease in the enrolled population. Eleven studies [19, 20, 22–25, 27, 28, 31–33] reported the number of patients who were excluded from the study due to SWE measurement failure for the reason of the presence of deep-seated lesion (greater than 8 cm from the skin); or with lesion near the heart and large blood vessels; or with lesion smaller than the size of the sampling box (smaller than 1 cm) for SWV measurement; or the patients’ inability to hold their breath properly. The percentage of SWE nonfeasible due to the technical limitations ranged from 1.2 to 26.3%, with the mean percentage of 12.7%. Eleven studies were conducted in Asia (7 in China, 3 in Korea, 1 in India), 3 in Europe, and 1 in North America (Canada).

**Table 1:** Main characteristics of the included studies

Author,year	Country	Elastography modality	Instrument	No.of patients	No.of liver lesions (Malignant)	Invalid measures(%)	Lesion types	Reference standard	Cut-off value (SWE/SWV ratio)
Cho et al., 2010 [19]	Korea	pSWE	Siemens, ACUSON S2000	51	60(43)	21.7%	HCC,CCC,LM,haemangioma	histopathology, CT/MRI	2 m/s
Davies et al., 2011 [21]	Holland	pSWE	Siemens, ACUSON S2000	37	45(10)	NR	haemangioma,LM	biopsy,CT/MRI	2.5 m/s (1.6)
Shuang-Ming et al., 2011 [20]	China	pSWE	Siemens, ACUSON S2000	116	128(68)	22.7%	HCC, CCC,LM, haemangioma,FNH,RN,FFS,FFC,abscess,adenoma,SNN	histopathology, CT/MRI	2.22 m/s
Yu et al., 2011 [23]	Canada	pSWE	Siemens, ACUSON S2000	89	105(41)	6.1%	HCC,LM,haemangioma,FNH,FFS,FFD,adenoma	biopsy, CT/MRI	1.9 m/s
Kapoor et al., 2011 [22]	India	pSWE	Siemens, ACUSON S2000	42	42(27)	12.5%	HCC,LM,haemangiomas,lymphomas,FNH,sarcoid,abscesses,FFS	biopsy	2.5 m/s
Kim et al., 2013 [26]	Korea	pSWE	Siemens, ACUSON S2000	74	101(73)	NR	HCC,CCC,LM, hemangioma	pathology, CT/MRI	2.73 m/s
Park et al., 2013 [27]	Korea	pSWE	Siemens, ACUSON S2000	47	47(39)	20%	HCC,CCC,LM,haemangioma,FNH	histopathology, CT/MRI	1.82 m/s
Zhang et al., 2014 [28]	China	pSWE	Siemens, ACUSON S2000	156	170(112)	9.7%	HCC,CCC,LM,hemangioma,FNH,RN	histopathology, CT/MRI,DSA	2.16 m/s
Guo et al., 2015 [25]	China	pSWE	Siemens, ACUSON S2000	134	134(55)	11.0%	HCC,CCC,LM,hemangioma,focal fatty degeneration,FFS,FNH, abscess	pathology, CT/MRI	2.13 m/s (1.37)
Lu et al., 2015 [24]	China	pSWE(Elast PQ)	Philips iu22	259	259(201)	1.2%	HCC,ICC,LM,hemangioma, FNH, CN	histopathology	13Kpa (1.3)
Wu et al., 2016 [29]	China	pSWE	Siemens, ACUSON S2000	46	55(27)	NR	HCC,LM,hemangioma,FFS,RN	pathology, CEUS, CT/MRI	2.22 m/s
Dong et al., 2017 [30]	China	pSWE(ElastPQ)	Philips EPIQ7	154	154(129)	NR	HCC,ICC,CHC,sarcoma,LM,AML,FNH,hemangioma,IPT	histopathology	2.06 m/s (1.67)
Wen-Shuo et al., 2016 [31]	China	2D-SWE(SuperSonic Imagine)	Aixplorer	221	229(164)	6.6%	HCC,ICC,CHC,LM,sarcomaneuroendocrine cancer,Unclassified cancer,FNH,hemangioma,angioleiomyolipoma,cavernous vascular tumor,abscess,Inflammatory pseudo-tumor,inflammation,solitary fibroma	histopathology,CEUS	Emax39.60Kpa/ Emean24.43 Kpa
Gerber et al., 2017 [32]	Germany	2D-SWE(SuperSonic Imagine)	Aixplorer	106	106(64)	24.3%	haemangioma,FNH, adenoma,FFS, RN,cholangiofibroma, HCC,CCC, LM	histopathology,CEUS, CT/MRI	Emean20.7 Kpa
Grgurevic et al., 2018 [33]	Switzerland	2D-SWE(SuperSonic Imagine)	Aixplorer	196	259(168)	26.3%	HCC,CCC,LM,haemangioma, FNH	biopsy, CT/MRI	Emean22.3 Kpa

*No* number, *HCC* hepatocellular carcinoma, *ICC* intrahepatic cholangiocellular carcinoma, *CCC* cholangiocarcinoma, *CHC* Combined hepatocellular carcinoma and intrahepatic cholangiocarcinoma, *LMN* liver metastasis, *FNH* focal nodular hyperplasia, *FFS* focal fatty sparing/ nodule of focal fatty sparing, *FFD* focal fat deposits, *FFC* focal fatty change, *SNN* solitary necrotic nodule, *RN* regenerative node, *CN* cirrhotic nodule, *AML* hepatic angiomyolipoma, *IPT* inflammatory pseudotumor, *CT* computer tomography, *MRI* magnetic resonance imaging, *CEUS* contrast enhanced ultrasound, MRI; *DSA* digital subtraction angiography, *NR* not reported

### Quality assessment of the included studies

**Fig. 2:**
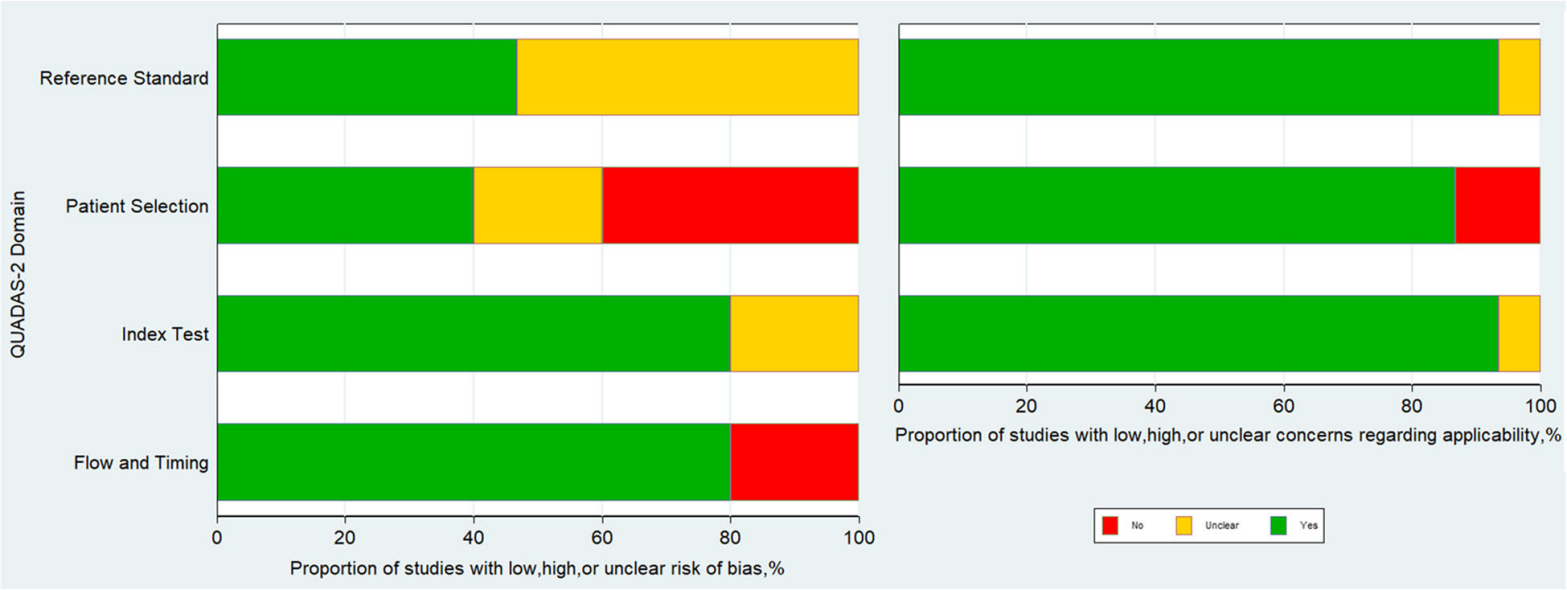
Quality assessment of the included studies according to Quality Assessment of Diagnostic Accuracy Studies-2 (QUADAS-2) criteria

The included studies conformed to the majority of the criteria of the QUADAS-2 and the overall quality of the studies was moderate (Fig. 2 and Additional file 1: Table S2). However, the risk of bias in patient selection was unclear in 3 studies [28, 29, 32] because it was not clear whether patients were consecutively enrolled in the cohort. Risk of bias in the index test was unclear in 3 studies [23, 25, 30] because they did not report whether SWE imaging was performed blinded to the reference standard. In 8 studies [19, 21, 25–30], the risk of bias in the reference standard was unclear because it was unclear whether the reference standard results were interpreted without knowledge of the measurement of SWE. The time interval between SWE imaging and the reference standard was not described in 3 studies [19, 20, 31].

### Pooled analysis of diagnostic accuracy and heterogeneity assessment

**Fig. 3:**
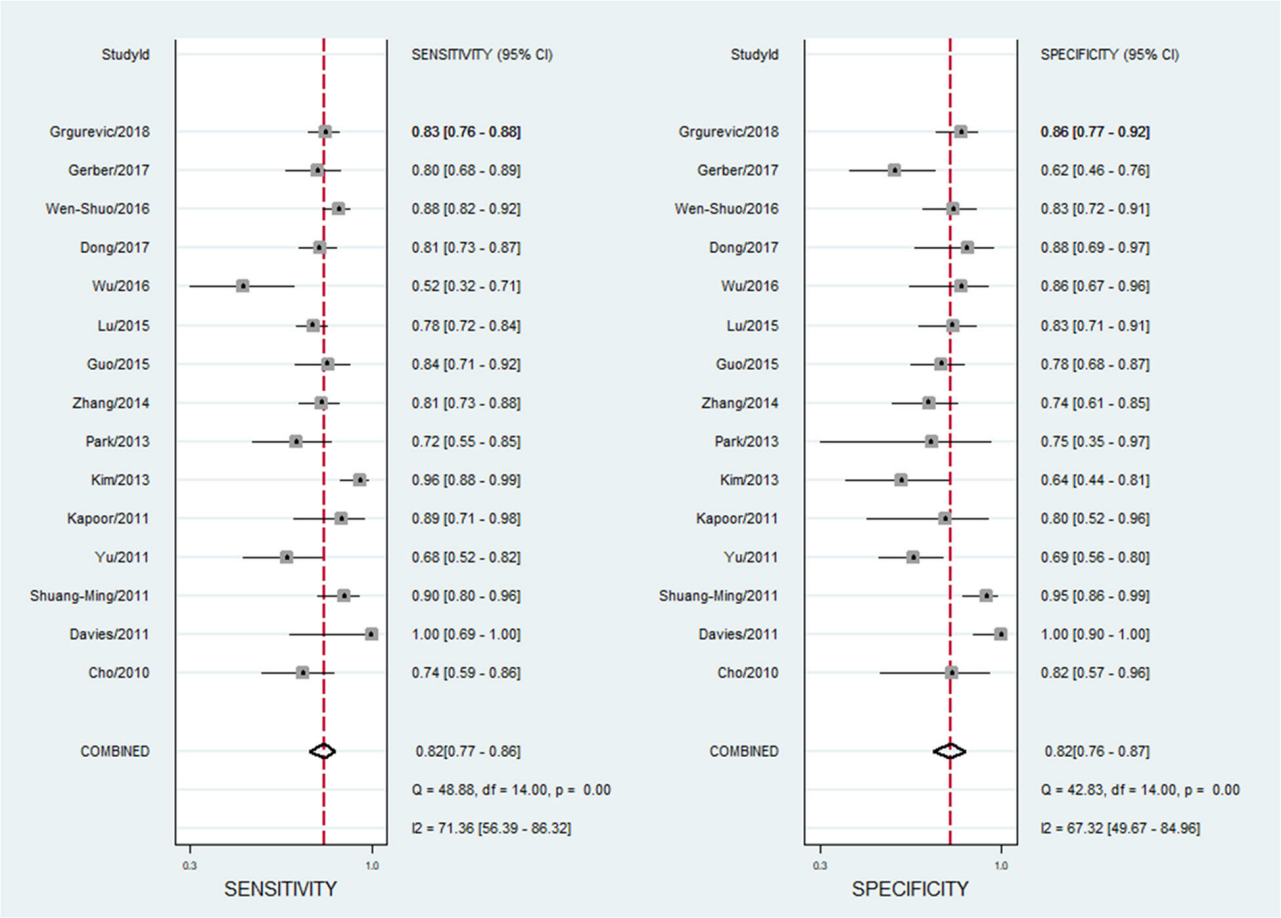
Sensitivity and specificity forest plots of shear wave elastography for differentiation of malignant and benign liver lesions. The pooled sensitivity and specificity of the SWE for differentiation of malignant and benign malignant liver lesions were 0.82 (95%CI: 0.77–0.86) and 0.82 (95%CI: 0.76–0.87), respectively

**Fig. 4:**
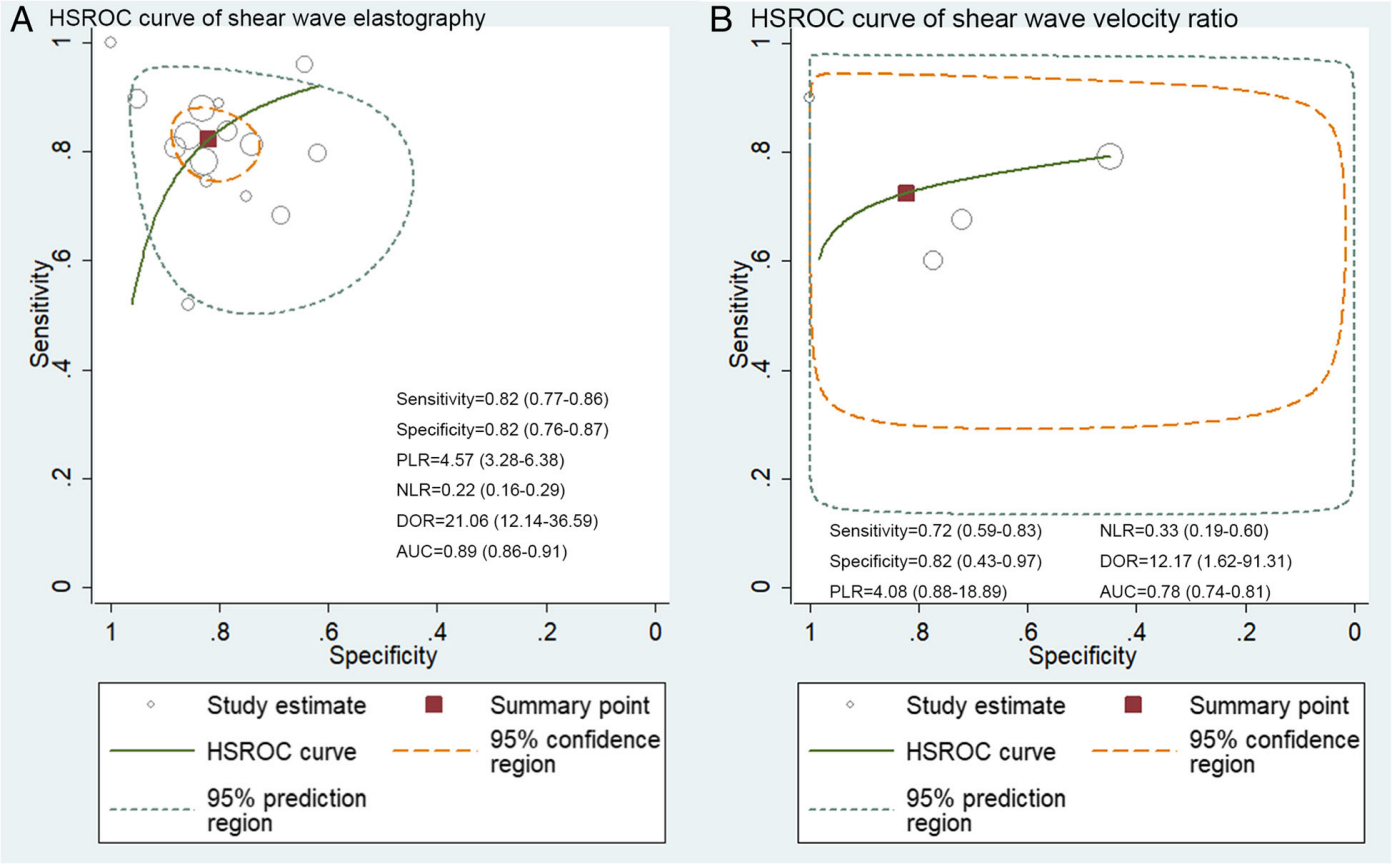
HSROC curve for differentiation of malignant and benign liver lesions of (**a**) shear wave elastography, (**b**) the shear wave velocity ratio (FLL to surrounding liver parenchyma). The AUC of the SWE and the SWV ratio for differentiation of malignant and benign malignant liver lesions were 0.89 (95%CI: 0.86–0.91) and 0.78 (95%CI: 0.74–0.81), respectively. (HSROC: Hierarchical summary receiver operating characteristic; FLL: focal liver lesion; PLR: positive likelihood ratio; NLR: negative likelihood ratio; DOR: diagnostic odds ratio; AUC: area under the curve)

The pooled sensitivity and specificity of SWE in differentiation of benign and malignant liver lesions were 0.82 (95% CI: 0.77–0.86) and 0.82 (95% CI: 0.76–0.87), respectively (Fig. 3). The positive likelihood ratio (PLR) and negative likelihood ratio (NLR) were 4.57 (95% CI: 3.28–6.38) and 0.22 (95% CI: 0.16–0.29), respectively; the summary diagnostic odds ratio (DOR) was 21.06 (95% CI: 12.14–36.59); and the area under the HSROC was 0.89 (95% CI 0.86–0.91) (Fig. 4a). There was statistically significant heterogeneity among the studies in pooling sensitivity (SEN) (*I*^2^ = 71.36%, *P* < 0.01), specificity (SPE) (*I*^2^ = 67.32%, *P* < 0.01), and DOR (*I*^2^ = 99.96%, *P* < 0.01). We performed a sensitivity analysis in which two studies [23, 27] (the cut-off values of SWV less than 2.0 m/s) were removed, whereas the results were not influenced seriously. We cannot separately calculate the diagnostic accuracy of SWE imaging in differentiation of liver lesions in patients with cirrhosis and non-cirrhosis, because the articles did not report the data or the data could not be extracted.

### Accuracy of SWV ratio for the differentiation of benign and malignant liver lesions

The pooled sensitivity, specificity, PLR, and NLR, of the SWV ratio (FLL to surrounding liver parenchyma) for the differentiation of malignant and benign liver lesions were 0.72 (95% CI: 0.59–0.83), 0.82 (95% CI: 0.43–0.97), 4.08 (95% CI: 0.88–18.89), and 0.33 (95% CI: 0.19–0.60), respectively; the summary DOR was 12.17 (95% CI: 1.62–91.31), and the area under the SROC was 0.78 (95% CI: 0.74–0.81) (Fig. 4b).

### Meta-regression and subgroup analyses

Univariate meta-regressions were performed to examine the sources of potential heterogeneity in sensitivity and specificity. The results showed that elastography modality, study location, gold standard, blinded interpretation of SWE, and attrition rate were significantly associated with the heterogeneity of sensitivity, whereas number of liver lesions, prevalence of malignant liver lesions, blinded interpretation of SWE, and attrition rate were significantly associated with the heterogeneity of specificity (Additional file 1: Figure S1). We performed subgroup analysis according to the elastography modality. The sensitivity of 2D-SWE was slightly higher compared with pSWE (84% vs. 82%, *P* < 0.01), whereas there was no significant difference in the specificity for the two modalities (*P* = 0.18). The sensitivity and the specificity of high attrition rate (≥10%) was higher than low attrition rate (<10%) (82% vs. 80%, P < 0.01; 81% vs. 78%, *P* < 0.05). Summary data stratified into several subgroups are shown in Table 2.

**Table 2:** Results of the meta-regression and subgroup analyses on shear wave elastography for differentiation of malignant and benign liver lesions

Covariates	Subgroup	No.of studies	Pooled SEN (95% CI)	*P* value	Pooled SPE (95% CI)	*P* value	Meta-Regression Joint *P* Value
modality	1-pSWE	12	0.82(0.76–0.87)	<0.01	0.83(0.77–0.89)	0.18	0.70
	0-2D-SWE	3	0.84(0.75–0.93)		0.79(0.66–0.92)		
location	1-Asian	11	0.82(0.77–0.88)	0.02	0.82(0.76–0.89)	0.05	0.99
	0- European or North America	4	0.82(0.72–0.92)		0.81(0.71–0.92)		
gold standard	1-histopathology	3	0.82(0.72–0.92)	0.02	0.85(0.73–0.96)	0.22	0.89
	0- histopathology and/or other	12	0.82(0.77–0.88)		0.81(0.75–0.88)		
number of lesions	1- ≥ 100	10	0.84(0.79–0.88)	0.06	0.80(0.73–0.86)	<0.01	0.10
	0-<100	5	0.76(0.66–0.87)		0.88(0.80–0.97)		
prevalence of malignant lesions	1- ≥ 50%	11	0.84(0.79–0.88)	0.14	0.81(0.74–0.88)	0.02	0.29
	0-<50%	4	0.75(0.63–0.87)		0.84(0.74–0.94)		
blinded	1-yes	11	0.82(0.77–0.88)	0.02	0.81(0.74–0.88)	0.02	0.79
	0-unclear	4	0.82(0.72–0.92)		0.85(0.75–0.95)		
attrition rate	1- ≥ 10%	7	0.82(0.77–0.87)	<0.01	0.81(0.74–0.88)	0.04	<0.01
	0-<10%	4	0.80(0.75–0.86)		0.78(0.69–0.87)		

*SEN* sensitivity, *SPE* specificity

### Assessment of clinical utility of SWE for differentiation of liver lesions

**Fig. 5:**
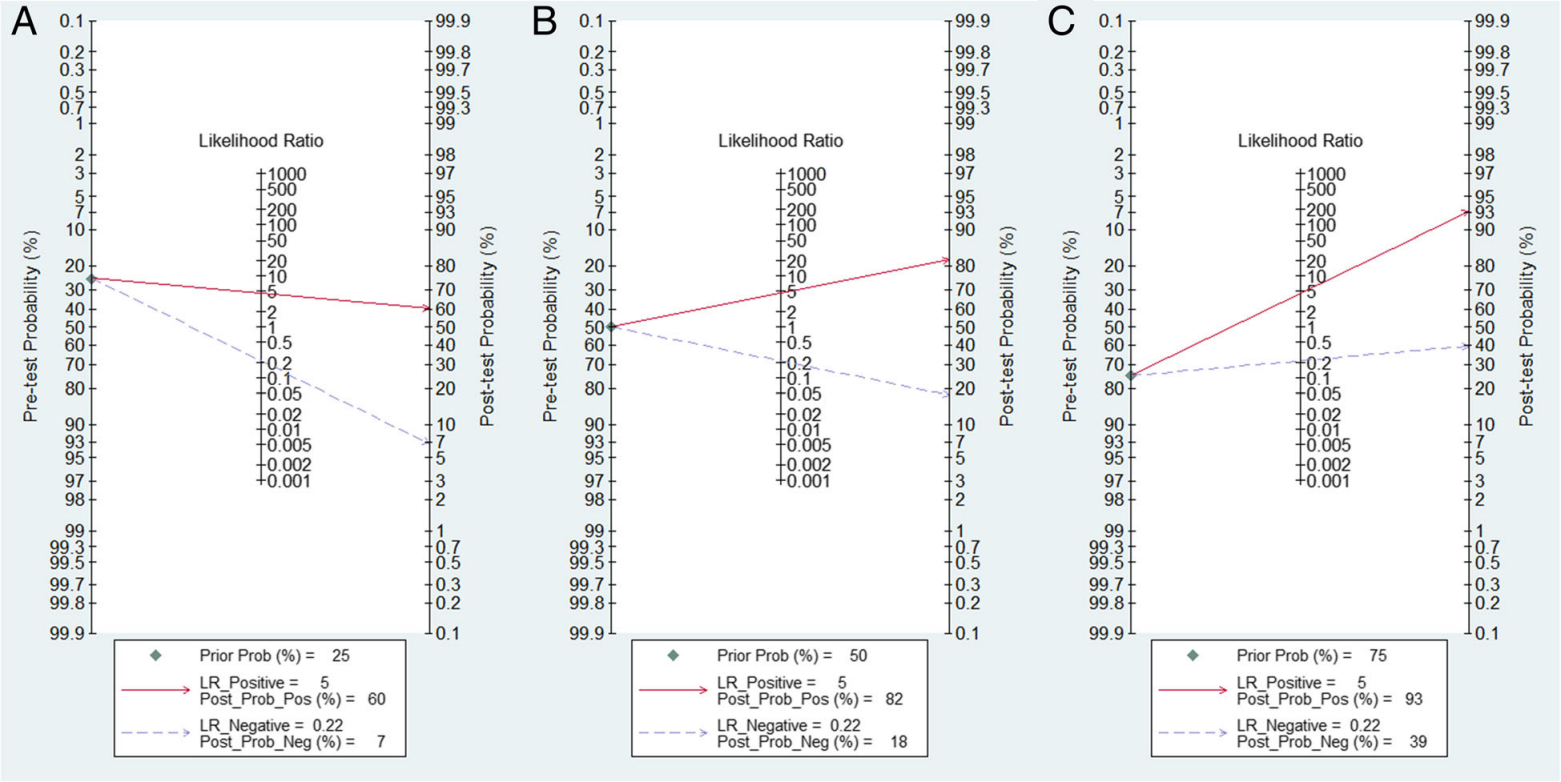
Fagan plot to evaluate the clinical utility of shear wave elastography for differentiation of liver lesions. (A) Pre-test probability = 25%; (B) Pre-test probability = 50%; (C) Pre-test probability = 75%. When the pre-test probability was 25%, with 60% probability of malignant disease following a positive measurement, and the probability reduced to 7% when a negative measurement occurred (Fig. **a**). When the pre-test probability was 50%, with 82% probability of malignant disease following a positive measurement, and the probability reduced to 18% when a negative measurement occurred (Fig. **b**). When the pre-test probability was 75%, with 93% probability of malignant disease following a positive measurement, and the probability reduced to 39% when a negative measurement occurred (**c**). (PLR: positive likelihood ratio; NLR: negative likelihood ratio)

The Fagan plot demonstrated that SWE imaging was fairly effective in distinguishing benign from malignant liver lesions when the pre-test probability was 50%, with 82% probability of malignant disease following a positive measurement, and the probability reduced to 18% when a negative measurement occurred (Fig. 5b). However, when the pre-test probability was 25%, probability was only 60% to differentiate malignant liver lesions correctly following a positive measurement of SWE imaging (Fig. 5a). In addition, the probability of a correct diagnosis rate reached 93% for malignant liver lesions following a positive measurement when the pre-test probability was 75%; nevertheless, the incidence of malignant lesions could reach 39% with a negative measurement (Fig. 5c).

### Publication bias

The Deeks’ funnel plot showed that the studies included in the meta-analysis were distributed symmetrically (*P* = 0.87, Additional file 1: Figure S2), indicating no clear evidence of publication bias.

## Discussion

Ultrasound elastography has been explored for application in many fields, it has shown good performance in the evaluation of liver fibrosis and the characterization of superficial organ lesions [34–36]. Similarly, focal liver lesions differ mechanically from surrounding tissues and show changes in their elasticity, with the tendency to increase stiffness in malignant lesions [8, 37]. Previous research have demonstrated that strain elastography is helpful for differentiation of benign and malignant liver lesions [38, 39], and enable us to distinguish accurately between HCC and metastatic adenocarcinoma [40]. SWE have been studied rencently to characterise focal liver lesions and have been proved to have certain clinical value in differential diagnosis [41, 42]. In this systematic evaluation and meta-analysis, we conducted an evidence-based summary of the performance characteristics of SWE in identifying malignant liver lesions.

The meta-analysis synthetic results indicate that SWE imaging has a high accuracy to discriminate malignant liver lesions from benign ones, with sensitivity, specificity, and AUC of 0.82 (95% CI: 0.77–0.86), 0.82 (95% CI: 0.76–0.87), and 0.89 (95% CI: 0.86–0.91), respectively. Furthermore, the results of the Fagan plot show that SWE imaging is valuable in differentiating liver lesions. When the pre-test probability was 50%, the correct diagnosis of malignant liver lesions increased to 82% after the SWE measurement was positive, whereas when the measurement was negative, malignant liver lesions were present in only 18% of patients. Therefore, it was reasoned that SWE imaging is promising and would play an important role in clinical practice. SWE imaging is easily and inexpensively integrated into the ultrasound systems and can be performed with one conventional probe so that the operator can visualize the liver directly, and the region of interest can be positioned manually at the specific location [43]. In addition, SWE imaging is rarely affected by ascites and obesity, because the generated shear waves originate inside the liver rather than from the body surfaces [44]. The results of a prior meta-analysis was also encouraging [45], with the sensitivity, the specificity, and the AUC of 0.86, 0.89, and 0.94, respectively, yet only 8 studies assessing diagnostic the performance of pSWE were included. Another meta-analysis [46] discussing the efficiency of SWE imaging for detecting malignant lesions of the liver also showed good results, with sensitivity, specificity and AUC of 0.82, 0.80 and 0.87, respectively; however, the 9 studies included were all to evaluate the diagnostic performance of pSWE while 2D-SWE studies were not included in this meta-analysis.

FLLs may occur on different liver backgrounds; shear wave velocity (SWV) of SWE imaging in the same type of focal lesion is variable with different surrounding parenchyma. Cirrhosis is an important cause of increased liver parenchyma stiffness. It is noteworthy that the liver is surrounded by a stiff, expandable capsule (Glisson’s capsule), so liver stiffness would increase following any increase in its volume caused by inflammation, cholestasis, or steatosis [44, 47]. Some viewpoints speculate that the ratio of SWV values (FLL to the surrounding liver parenchyma) can more accurately differentiate malignant lesions [48]. However, our meta-analysis found that the cut-off value of the SWV/elasticity in FLL showed superior performance compared to the cut-off value of the SWV ratio, with an AUROC of 0.89 vs. 0.78. The diagnostic performance for the sum of SWV values (FLL and the surrounding liver parenchyma) to differentiate liver lesions is reported in only one article [27], with an AUROC of 0.853.

Despite the results of our study showing promising results, there were still some technical limitations in SWE imaging. First, the maximum detection depth of SWE is limited. The transmission of an acoustic radiation impulse was allowed only up to 10 cm from the skin in pSWE, owing to safety concerns [49]. The maximum detection depth of 2D-SWE would be deeper than pSWE but would depend on the type of instrument and probe. It is difficult to detect the shear wave of a deep-seated lesion because of high attenuation of the signal as it propagates, whereas a shear wave attenuates more slowly due to the Mach cone’s effect in 2D-SWE. Second, the shear wave’s speed that SWE measured was susceptible to motion-related factors, and the accuracy of the results decreased when the lesion was close to the heart and large vessels, or when patients had poor breath-holds. Third, there are wide ranges of stiffness values for focal liver lesions, and some values overlap between the benign and malignant lesions [50]. It seems likely that malignant liver lesions are usually stiffer than benign ones, especially for tumours with pronounced desmoplastic stroma reaction such as intrahepatic cholangiocellular carcinoma, but internal hemorrhage or necrosis in malignant lesions would decrease stiffness. The stiffness of some benign lesions with a high proportion of fibrous tissue such as focal nodular hyperplasia can increase due to fibrous septa and central scar [34]. Finally, there is a high potion of the measurement failure among the included studies (from 1.2 to 26.3%), the diversity of attrition rate may be related to the patient inclusion criteria of the original studies and the proficiency of the operator. In general, patients with FLLs in the right liver lobe and a proximal edge located < 7 cm from the body’s surface would be easily detected, and patients with successful measurement had a lower body mass index (BMI) as compared to patients in which SWE measurement failed. A few studies demonstrate that the performance of SWE for FLL characterization appears limited due to the overlap of the stiffness values between the benign and malignant lesions [50, 51].

There are several potential limitations in our study that should be taken into consideration. First, a considerable amount of heterogeneity was detected among the included studies. Our subgroup analyses found that potential sources of heterogeneity included elastography modality, study location, prevalence of malignant liver lesions, blinded interpretation of SWE, attrition rate, and several other differences, which were unrecorded in these studies, might also contribute to the heterogeneity. Second, because of the limited number of included studies, only 3 studies assessed 2D-SWE, and our study included some relatively small samples of studies; large-sample and multicenter studies on the different kinds of elastography modality in different liver parenchymal settings (such as cirrhosis and non-cirrhosis) are still needed. Third, the summary measurement of diagnostic accuracy pooled the optimal results from each study, with diverse cut-off values, which also may result in overestimation of the performance of SWE imaging. Nevertheless, in clinical practice, one criterion of cut-off value would be required for differentiating liver malignant lesions. Fourth, original studies included in our meta-analysis did not provide size-stratified subgroup analysis results which in turn make it hard to identify appropriate size of lesion to do SWE imaging. Future studies are needed to do subgroup analysis according to tumor size to investigate the proper size. In addition, only English-language articles were included in our study; thus, language bias may have influenced the results. Therefore, considering these limitations, our findings should be interpreted with caution.

## Conclusions

In conclusion, our meta-analysis suggests that SWE imaging has favorable diagnostic value for differentiating malignant liver lesions from benign ones. SWE imaging is a promising method undergoing rapid development [52], which could give additional important information to conventional ultrasound, with high sensitivity and specificity in differential diagnosis of liver lesions. It should be emphasized that the SWE imaging assessment of a liver lesion should be interpreted in the context of the patient’s clinical background. Future large-scale studies are required to evaluate the performance of SWE imaging in differentiation of malignant liver lesions and to determine an optimal cut-off value.

## Additional file

Additional file 1: **Table S1.** Characteristics of the diagnostic performance of SWE imaging in included studies. **Table S2.** Quality assessment with QUADAS-2. **Figure S1.** Univariate meta-regression and subgroup analyses for sensitivity and specificity of SWE imaging. **Figure S2.** Deeks’ funnel plot asymmetry test for publication bias. (DOCX 1237 kb)

## Abbreviations

2D-SWE: two-dimensional shear wave elastography
ARFI: acoustic radiation force impulse
AUC: area under the ROC curve
BMI: body mass index
CEUS: contrast-enhanced ultrasound
FLL: focal liver lesion
HSROC: hierarchical summary receiver operating characteristic
NAFLD: Non-alcoholic fatty liver disease
pSWE: point shear wave elastography
SWE: shear wave elastography
SWV: shear wave velocity
TE: transient elastography
