# Supplementary material for: Diagnostic effect of shear wave elastography imaging for differentiation of malignant liver lesions: a meta-analysis
Source: BMC Gastroenterol. 2019 Apr 25;19:60. doi: 10.1186/s12876-019-0976-2 (PMC6485138; doi:10.1186/s12876-019-0976-2)
Supplement: Supplementary file 1 — Table S1. Characteristics of the diagnostic performance of SWE imaging in included studies. Table S2. Quality assessment with QUADAS-2. Figure S1. Univariate meta-regression and subgroup analyses for sensitivity and specificity of SWE imaging. Figure S2. Deeks’ funnel plot asymmetry test for publication bias. (DOCX 1237 kb) [file 12876_2019_976_MOESM1_ESM.docx]

**Supplementary Table 1 Characteristics of the diagnostic performance of SWE imaging in included studies**

| Author,year | Design | Nodule size (Mean/range)(cm) | Cirrhosis(%) | CLD(% ) | TP | FP | FN | TN |
| --- | --- | --- | --- | --- | --- | --- | --- | --- |
| Cho et al, 2010 (19) | P | HCC:3.6/1.6-7.9,CCC:3.5/0.7-8.4 ,LM and haemangiomas: 1.6/0.8-3.0 | NR | 43.1% | 32 | 3 | 11 | 14 |
| Davies et al, 2011 (21) | P | Haemangiomas: 2.16/0.7-7.0, LM: 4.14/1.4-9.0 | NR | NR | 10 | 0 | 0 | 35 |
| Shuang-Ming et al, 2011 (20) | P | malignant:3.3/1.1-10.3,benign:3.1/1.2-7.4 | NR | 32.8% | 61 | 3 | 7 | 57 |
| Yu et al, 2011 (23) | P | 2.77/1.0-13.0 | 27.0% | 27.0% | 28 | 20 | 13 | 44 |
| Kapoor et al, 2011 (22) | P | NR | 14.3% | NR | 24 | 3 | 3 | 12 |
| Kim et al, 2013 (26) | P | ≥1.56 | NR | 27.0% | 70 | 10 | 3 | 18 |
| Park et al, 2013 (27) | P | ≥2.0 | NR | NR | 28 | 2 | 11 | 6 |
| Zhang et al, 2014 (28) | P | ≥1.0 | NR | NR | 91 | 15 | 21 | 43 |
| Guo et al, 2015 (25) | P | ≥1.0 | NR | NR | 46 | 17 | 9 | 62 |
| Lu et al, 2015 (24) | P | NR | NR | NR | 157 | 10 | 44 | 48 |
| Wu et al, 2016 (29) | P | NR/1.1-7.2 | NR | NR | 14 | 4 | 13 | 24 |
| Dong et al, 2017 (30) | P | 5.5/1.5-14.5 | 39.6% | 65.6% | 104 | 3 | 25 | 22 |
| Wen-Shuo et al, 2016 (31) | P | 4.6/1.0-15.4 | NR | 49.3% | 144 | 11 | 20 | 54 |
| Gerber et al, 2017 (32) | P | malignant:2.7/1.0-8.7, benign:3.2/1.1-11.8 | NR | NR | 51 | 16 | 13 | 26 |
| Grgurevic et al, 2018 (33) | P | 3.98/0.4-15.0 | 27.0% | NR | 139 | 13 | 29 | 78 |

SWE: shear wave elastography; P: Prospective; CLD: chronic liver disease; TP: true positive; FP: false positive; FN: false negative; TN: true negative; NR: not reported

**Table 2 Quality assessment with QUADAS-2**

| Reference | Risk of bias | | | | Applicability concerns | | |
| --- | --- | --- | --- | --- | --- | --- | --- |
|  | Patient selection | Index test | Reference standard | Flow and timing | Patient selection | Index test | Reference standard |
| Cho, 2010 (19) | Low | Low | Unclear | High | Low | Low | Unclear |
| Davies, 2011 (21) | Low | Low | Unclear | Low | High | Low | Low |
| Shuang-Ming, 2011(20) | High | Low | Low | High | Low | Low | Low |
| Yu, 2011 (23) | Low | Unclear | Low | Low | Low | Unclear | Low |
| Kapoor, 2011 (22) | Unclear | Low | Low | Low | Low | Low | Low |
| Kim, 2013 (26) | Low | Low | Unclear | Low | Low | Low | Low |
| Park, 2013 (27) | Low | Low | Unclear | Low | Low | Low | Low |
| Zhang, 2014 (28) | Unclear | Low | Unclear | Low | Low | Low | Low |
| Guo, 2015 (25) | High | Unclear | Unclear | Low | Low | Low | Low |
| Lu, 2015 (24) | High | Low | Low | Low | Low | Low | Low |
| Wu, 2016 (29) | Unclear | Low | Unclear | Low | Low | Low | Low |
| Dong, 2017 (30) | High | Unclear | Unclear | Low | Low | Low | Low |
| Wen-Shuo, 2016 (31) | High | Low | Low | High | High | Low | Low |
| Gerber, 2017 (32) | High | Low | Low | Low | Low | Low | Low |
| Grgurevic, 2018 (33) | Low | Low | Low | Low | Low | Low | Low |

QUADAS-2: Quality assessment of diagnostic accuracy studies-2; Low: Low Risk; High: High Risk; Unclear: Unclear Risk

**Supplementary Figure 1 Univariate meta-regression and subgroup analyses for sensitivity and specificity of SWE imaging**

**
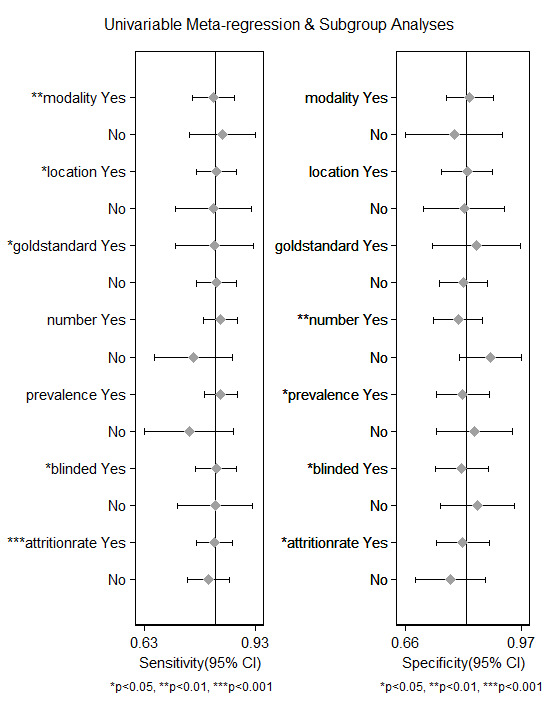
**

Elastography modality, study location, gold standard, blinded interpretation of SWE imaging, and attrition rate were significantly associated with the heterogeneity of sensitivity (all *p*s<0.05); number of liver lesions, prevalence of malignant liver lesions, blinded interpretation of SWE imaging, and attrition rate were significantly associated with the heterogeneity of specificity (all *p*s<0.05).

**Supplementary Figure 2 Deeks’ funnel plot asymmetry test for publication bias.**

**
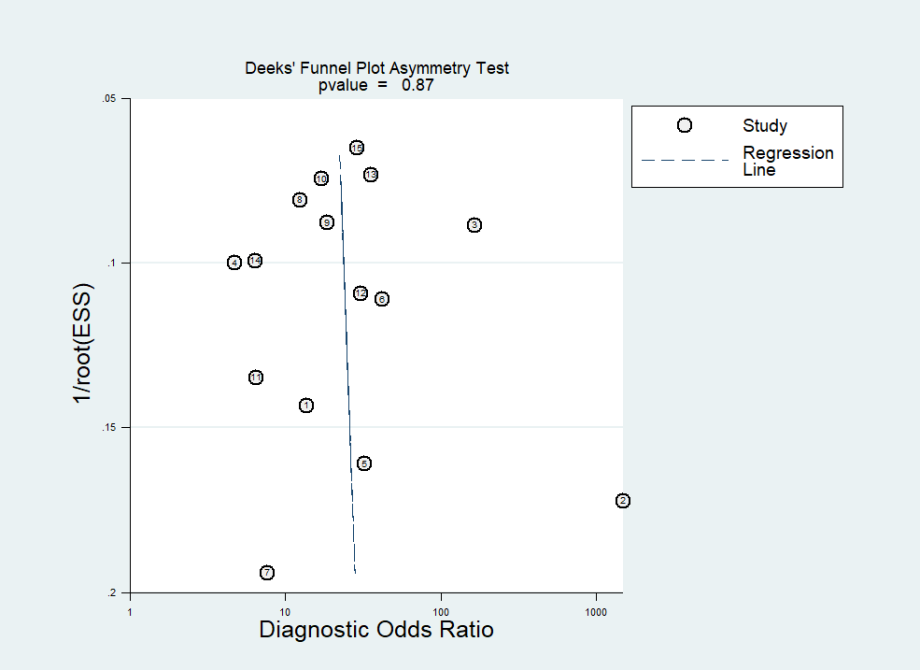
**

Deeks’ funnel plot indicate that no signifcant bias was found (*P*=0.87).
